# Mixed-species bacterial swarms show an interplay of mixing and segregation across scales

**DOI:** 10.1038/s41598-022-20644-3

**Published:** 2022-10-03

**Authors:** Gal Natan, Vasco M. Worlitzer, Gil Ariel, Avraham Be’er

**Affiliations:** 1 Zuckerberg Institute for Water Research, The Jacob Blaustein Institutes for Desert Research, Ben-Gurion University of the Negev, Sede Boqer Campus, 84990 Midreshet Ben-Gurion, Israel; 2 Department of Mathematics, Bar-Ilan University, 52900 Ramat-Gan, Israel; 3 Department of Physics, Ben-Gurion University of the Negev, 84105 Beer-Sheva, Israel

**Keywords:** Biological fluorescence, Biophysics, Physics

## Abstract

Bacterial swarms are a highly-researched example of natural active matter. In particular, the interplay between biological interactions and the physics underlying the swarming dynamics is of both biological and physical interest. In this paper, we study mixed swarms of *Bacillus subtilis* and *Pseudomonas aeruginosa*. We find intricate interactions between the species, showing both cooperation and segregation across different spatial and temporal scales. On one hand, even though axenic colonies grow on disparate time scale, an order of magnitude apart, the two-species swarm together, forming a single, combined colony. However, the rapidly moving populations are locally segregated, with different characteristic speeds and lengths (or cluster sizes) that depend on the ratio between the species. Comparison with controlled mutant strains suggest that both the physical and known biological differences in species characteristics may not be enough to explain the segregation between the species in the mixed swarm. We hypothesize that the heterogeneous spatial distribution is due to some mechanism that enables bacteria to recognize their own kind, whose precise origin we could not identify.

## Introduction

Collective motion of individuals in a group is a phenomenon observed in nature at different scales^1,2^. Among all species, bacteria provide an excellent model for analyzing the behavior of large populations because they can be easily observed, their movement can be accurately recorded, and they are amenable to genetic and environmental manipulations. In nature, bacteria grow in a variety of habitats. In most cases, several species, or variants of the same species, occupy the same niche, creating a heterogeneous population with a diverse range of interactions between them (e.g.,^3,4^). It has been suggested that population heterogeneity could enable bacteria to better exploit the local environment as well as to optimize exploration of new territories^5,6^. On the other hand, different species that compete over the same limited resources, may result in antagonistic interactions such as secretion of chemicals that impact the bacterial physiology and differentiation of the rival species, inhibit biofilm formation and release toxins^7,8^.

In this paper, we focus on bacterial swarms, which are colonies composed of elongated rod-shaped cells that migrate rapidly on semi-solid surfaces, powered by flagella that propel and create thrust^5,6,9–19^. In some species the cells produce biosurfactants to ease the motion by lowering the surface tension of the medium in which the cells move^20^. While in the swarm state, cells gain benefits; these include better expansion and dissemination rates^21–23^, improved access to nutrients and oxygen^10^, as well as resistance to antibiotics or other adverse materials^24^. The motion of the cells is characterized by coherent flows and jets that form dynamic clusters that split and merge^6^. Swarming, as a phenomenon, is not species-specific. Examples of known swarm species include *Bacillus subtilis*^5^, *Escherichia coli*^18^, *Serratia marcescens*^25,26^, *Proteus mirabilis*^27^, *Paenibacillus dendritiformis*^28,29^, *Pseudomonas aeruginosa*^11,30^ and *Salmonella*^31^.

Most of the literature on swarming bacteria is focused on *homogeneous* systems, in particular single-species (axenic) cultures. Working with a single species is typically done in order to understand basic properties of swarming in a specific chosen system and uncover the principle underlying mechanisms. In addition, heterogeneous systems pose new technical difficulties, as cells need to be distinguished throughout the experiments. Nevertheless, recent studies on the microscopic dynamics of *heterogenous* swarm systems was done by mixing two swarming strains of the same species. Peled et al.^31^ considered the mixing of two strains of *B. subtilis* that differ in cell aspect ratio (cell length). They found that the mixed swarm is not spatially segregated into long-cells/short-cells regions. In fact, the opposite phenomenon was observed, as locally, long cells act as nucleation cites, around which aggregates of short, rapidly moving cells can form, resulting in enhanced swarming speeds. Anyan et al.^11^ showed that single-strain swarms of a pili-defective mutant (absence of type-IV pili) of *P. aeruginosa* behave differently compared to the wild-type (WT), as they expand faster due to different cell–cell interactions. When the two strains (WT and pili-defective) were mixed in the same colony, a spatial separation was observed. Benisty et al.^32^ showed that swarm colonies of WT *B. subtilis*, exposed to low levels of kanamycin, spontaneously form a sub-population of motility-defective cells that self-segregates; they also showed that a mixed swarm of WT cells and immotile cells (with no exposure to kanamycin) behave similarly. Zuo et al.^33^ showed that a mixed swarm colony, composed of two strains of *E. coli* that differ in motility, forms intricate dynamics. In particular, it was shown that motility heterogeneity can promote spatial segregation of subpopulations via a dynamic motility selection process. Recently, Jose et al.^19^ mixed *B. subtilis* and *Serratia marcescens* and studied the dynamics of a mixed-species swarm colony. The main point in their study was to demonstrate that mixed species can indeed form robust swarming. Those swarms were thick, preventing microscopic tracking of each of the species separately, hence forming a relatively poor model for 2D active matter. All in all, heterogenous swarm colonies composed of strains of the same species provided intricate, sometimes conflicting results. Moreover, mixing two species in the same swarm colony and studying its precise dynamics at the microscopic scale is still lacking.

In this work, we study the dynamics of mixed-species swarm colonies. In particular, we are interested in quantifying in which aspects mixed-species swarms are different from same-species mixtures with similar physical characteristics. The physics underlying the swarming phenomenon is blind to the biological species. Hence, the differences between same and mixed-species swarm shed light on fundamental questions regarding the interplay between physical and biological interactions within bacterial swarms.

In order to address these questions, we mix *B. subtilis* with *P. aeruginosa*. The latter is a significantly slower swarmer and has a smaller cell size. Both species have been well-studied axenically as swarmers, but were mixed in the past in non-swarming setups, showing a variety of biological inhibitions^7^. The latter makes the system interesting as it increases the plausible types of interaction between the two species. In order to distinguish between the species, each was labelled fluorescently using a different color.

**Figure 1:**
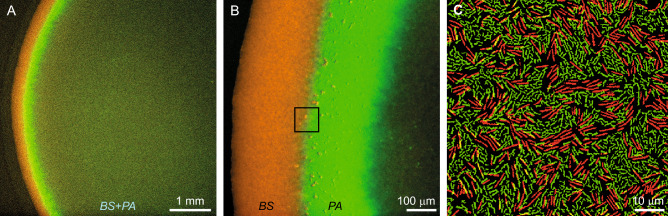
A mixed swarm of WT *B. subtilis* (red) and WT *P. aeruginosa* (green)—fluorescence microscopy images. (**A**) Low magnification shows a macroscopic view of the colony. Even though axenic *P. aeruginosa* grow an order of magnitude slower than axenic *B. subtilis* colonies, the two species grow together, forming a single, combined colony. (**B**) Larger magnification emphasizes that the two species are macroscopically separated, forming slightly overlapping bands. The boundary between the bands is not sharp. (**C**) A microscopic view of the intermediate region between the bands shows that the species are mixed, but not homogeneously. Local segregation into clusters with a scale of 10–20 µm can be seen. The black square in (**B**) shows the zoom-in region depicted in (**C**).

Our main finding is that the two species exhibit macroscopic spatial separation, and in the mixed zone, arrested microscopic segregation (Fig. 1 and Movie S1). By arrested segregation, we mean that, on average, each cell is more likely to be closer to other cells of the same species. However, the two species are not fully segregated and there is no inhibition region between them. In the mixed zone, the speeds of the two species were found to be different, pointing to weak hydrodynamic interactions and weak speed alignment.

## Methods

### Species and growth protocol

The bacterial species used in this study were *Bacillus subtilis* NCIB 3610 “wild-type” and *Pseudomonas aeruginosa* PAO1C “wild-type”. Both species were fluorescently labelled using fluorescent proteins to distinguish between them in a mix. *B. subtilis* was labelled red (strain number 4847; pAE1222-LacA-Pveg-R0_mKate, mls), and *P. aeruginosa* was labelled green (miniTn7 site)^11^. In both, the fluorescence was stable throughout the experiment and did not show any cost of carriage or photobleaching. Antibiotics, at a concentration of 100 μg/ml was added to frozen stocks and to hard-agar (2% and 25 g/l Luria Bertani (LB)) isolation plates; streptomycin to *B. subtilis* and gentamycin to *P. aeruginosa*. Experiments were performed without additions of antibiotics and fluorescence was completely stable.

Isolated colonies from hard-agar plates were cultured separately (each species on its own) in a 2 ml LB liquid medium (15 ml tubes) and incubated at 200 rpm and 30 °C for 17 h. The overnight cultures were mixed at different ratios in small vials and vortexed for few seconds before inoculation to the swarm plates; a small 4-µl drop at the center of a swarm plate. Swarm plates are standard 8.8 cm Petri-dishes filled with 15 ml of molten 0.5% agar supplemented with LB. Swarm plates were prepared 20 h prior to inoculation and aged at 22 °C and 40% relative humidity (RH), then 8 min in the flow hood. After inoculation, swarm plates were incubated at 30 °C and 95% RH until the diameter of the swarm colony was 4 cm, then observed under an optical microscope. These particular conditions were chosen to equally meet the favourable growth conditions of the two species. A variety of strains/mutants/variants were used in the study. Kindly see Table 1 for details.

**Table 1:** A list of all strains used in this work, their main feature, and origin.

Species name	Strain name	Mutation	"Color"	Relevant characteristics	Source
*B. subtilis* 3610	WT	–	–	–	D B Kearns^a^
*B. subtilis*	WT-Green	amyE::PvegR0 sfGFP spec	Green	Labelled	A Eldar^b^
*B. subtilis*	WT-Red	pAE1222-LacA-Pveg-R0_mKate, mls	Red	Labelled	A Eldar^b^
*B. subtilis*	DS1102 (1043)	srfAA::Tn10 spec	–	No surfactant	D B Kearns^c^
*B. subtilis*	DS1470	swrA::tet amyE::Physpank-swrA spec	–	Slow, deleted for the native copy of swrA	D B Kearns^d^
*B. subtilis*	DS1470-Red	swrA::tet amyE::Physpank-swrA spec pAE1222-LacA-Pveg-R0_mKate, mls	Red	Slow, deleted for the native copy of swrA, Labelled	A Eldar*
*B. subtilis*	DS858-Green	minJ::tet amyE::Pveg R0 sfGFP spec	Green	Elongated, Labelled	A Eldar^b^
*P. aeruginosa* PAO1C (ATCC 15692)	WT	–	–	–	J D Shrout^e^
*P. aeruginosa*	WT-GFP	PAO1C::miniTn7 gfp2; Cm, Gm	Green	Labelled	J D Shrout^e^
*P. aeruginosa*	∆pilA-GFP	PAO1C-∆pilA-gfp	Green	No type-IV Pili Labelled	J D Shrout^e^
*P. aeruginosa*	∆fliM-GFP	PAO1C ∆fliM miniTn7 gfp3; Tcr, Kmr, Smr	Green	Immotile (no polar flagella) Labelled	J D Shrout^e^
*P. aeruginosa*	102	∆lasR	–	Quorum sensing defective	A Eldar^f^
*P. aeruginosa*	680	∆lasR attTn7::pA1/04/03-gfp(ASV)	Green	Quorum sensing defective Labelled	A Eldar^g^
*P. aeruginosa*	103	∆rhlR	–	Quorum sensing defective	A Eldar^f^
*P. aeruginosa*	104	∆lasR ∆rhlR	–	Quorum sensing defective	A Eldar^f^
*P. aeruginosa*	847	pUB-paGFP mexT-mutation	Green	Quorum sensing rhl enhanced Labelled	E Banin^h^
*P. aeruginosa*	∆pel-∆psl-GFP	∆pel ∆psl pMRP9-1 GFP	Green	EPS deficient Labelled	M R Parsek^i^

*This work, courtesy of A Eldar.

^a^Kearns. Nat Rev Microbiol. 8, 634–644 (2010).

^b^Peled et al. PRE. 103, 032413 (2021).

^c^Kearns et al. Mol Microbiol. 52, 357–369 (2004).

^d^Ilkanaiv et al. PRL. 118, 158002 (2017).

^e^Anyan et al. PNAS. 111, 18013–18018 (2014).

^f^Davies et al. Science. 280, 295–298 (1998).

^g^Xavier et al. Mol. Microbiol. 79, 166–179 (2011).

^h^Oshri et al. ISME J. 12(10), 2458–2469 (2018).

^i^Colvin et al. Environ. Microbiol. 14(8), 1913–1928 (2012).

### Microscopy

An epifluorescence optical microscope Axio Zen 16 (Zeiss), hooked to a camera (Axiocam 506 mono), was used to follow the expansion and dissemination of each species across the colony. The field of view in the microscope is large, up to about 1″ × 1″, but individual fluorescence cells are still resolved. Both species were tracked by two different channels, *B. subtilis* in a red channel and *P. aeruginosa* in a yellow channel (a yellow channel was used instead of a green one as the excitation light of the GFP setup destroys cell motility). These two channels were superimposed.

High resolution microscopy was used for studying the dynamics of the cells. We used an Optosplit II, Andor device, hooked to a Zeiss Axio Imager Z2 microscope and a 63 × LD lens. The system splits a dually excited image (Ex 59026x, beam splitter 69008bs, and Em 535/30; 632/60) on a NEO camera (900 × 1800 and 50 fps) in order to generate two simultaneous but separate fields of view, i.e., green (called green but may be considered yellow) and red, that are then merged again following post processing. In general, the *B. subtilis* (labelled red) appear on the left panel, and the *P. aeruginosa* (labelled green) appear on the right panel. Movies streamed directly to the hard disk, resulting in a sequence of thousands of images per experiment.

### Data analysis

Images from the two fluorescence channels were pre-processed using the methods described in^34^. The collective motion of the cells (each species by itself) was studied using optical flow (OF). The analysis was done using MATLAB.

## Main results

### Axenic colonies

Each species was grown on its own in a swarm plate under canonical conditions (25 g/l LB and 0.5% agar) starting from a 4-µl drop of an overnight culture. The *B. subtilis* was fluorescently labelled red and *P. aeruginosa* was fluorescently labelled green^11^ (to assist later in the mixed assays). *B. subtilis* swarm colonies lag ~ 3 h and then expand very fast, covering the entire plate within 3 more hours. Microscopically, the motion of the cells in the swarm is of the order of 10’s of µm/s. *B. subtilis* form a monolayer of cells and their density changes along the distance from the colony center to the exterior, with typically less cells at the edge and more towards the center^35^. On the other hand, *P. aeruginosa* lag much longer (~ 6 h), and when they eventually start spreading, they do so at much slower speeds; they cover the entire plate only several days after inoculation. Similarly, on the microscopic scale, although they secrete surfactant and are flagellated, their average microscopic speed is practically close to 0 µm/s, where only a few of the cells are observed to be moving very slowly among the immobile crowd (why *P. aeruginosa* swarm slower compared to *B. subtilis* is not known; it might be related to the power of flagella and type of surfactant). *P. aeruginosa* form a thicker layer of cells (~ 3 layers) with no obvious variation in density along the colonial diameter. Overall, while both species swarm and eventually spread through the entire plate, they do so on disparate time scales, both in the local and global dynamics.

### Wild-type *B. subtilis* mixed with wild-type *P. aeruginosa*

Swarm colonies were grown from homogenously-mixed cultures. Two wild-type (WT) strains were mixed, each of the species was labelled fluorescently (*B. subtilis* is fluorescently labelled red and *P. aeruginosa* is fluorescently labelled green). Macroscopically, the mixed colony exhibits two fluorescent fronts, indicating that the swarm is heterogenous on the colony-scale. Such fronts of dense differential populations were not seen in our axenic colonies in the current study (note that similar dense fronts were observed in other *B. subtilis* swarm studies^36^ as well as in *Paenibacillus dendritiformis* past studies^28^). The *B. subtilis* forms the precursor front of the swarm (red band in Fig. 1A, B), while the *P. aeruginosa* forms a second front (green), right behind the red one. On the microscopic scale, the intermediate region between the fronts was inhabited by the two species but with much lower densities and lower activity, which is different from the case of both species grown axenically. Zooming in on the boundary between the two fronts (Fig. 1C and Movie S1), we see that the species are not well-mixed also on the microscopic scale. Overall, the expansion speed of the mixed colony (the precursor front) is half the speed of the axenic *B. subtilis* colony, indicating that the *B. subtilis* expand slower but that *P. aeruginosa* expand dramatically much faster (as they barely expand without the presence of *B. subtilis*).

**Figure 2:**
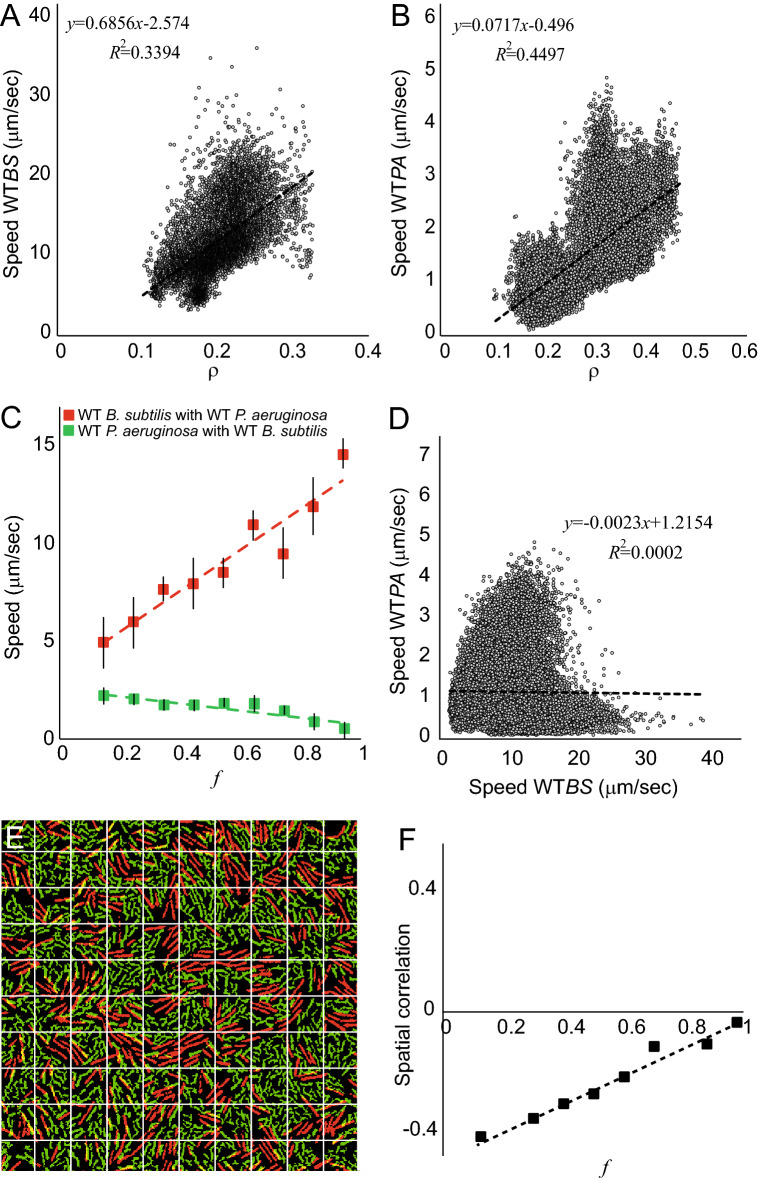
Dynamics analyses of mixed WT swarm colonies. (**A**) The speed of WT *B. subtilis* as a function of *ρ* (pooled over all *f*). The speed increases with the total cell density. (**B**) The speed of WT *P. aeruginosa* (pooled over all *f*)*.* The speed increases with the total cell density. (**C**) The average speed of the WT *B. subtilis* (red) is an increasing function of the density ratio *f* at fixed area fraction *ρ* = 0.25. The average speed of the WT *P. aeruginosa* (green) is a decreasing function of the density ratio *f* (the partial density of *B. subtilis*). The average is taken over different experiments and the error bars are standard deviations between experiments. Dashed lines are linear regressions. (**D**) The speed of *P. aeruginosa* as a function of the speed of *B. subtilis*, pooled overall *ρ* and *f*, shows no correlation. (**E**) Dividing the frame into equally-sized bins, then (**F**) calculating the spatial correlation between the area fraction of the two species as a function of *f*. The dashed line is a linear regression.

The microscopic bacterial speeds of each of the species was measured as a function of the total surface cell-density, *ρ*, defined as the average area fraction occupied by cells, and as a function of the partial WT *B. subtilis* ratio among all cell, *f*. For instance, *ρ* = 0 means that no cells are present, *ρ* ~ 0.9 surface is the maximal crowding, *f* = 1 means only *B. subtilis* and *f* = 0 only *P. aeruginosa*. Figure 2A, B show that the speed of each of the species increases with *ρ* as expected for swarm systems. Each data point corresponds to a specific *ρ* at a variety of *f*. In Fig. 2A we chose all data points with *f* > 0.5 and in Fig. 2B we chose all data points with *f* < 0.5 to reduce the influence of the competing species and enhance the dependence on *ρ*. However, Fig. 2C shows that for fixed *ρ* (here, *ρ* = 0.25) the average (over many experiments for the same *f*) microscopic speed of *B. subtilis* increases linearly with *f* whereas the average microscopic speed of *P. aeruginosa* decreases linearly with *f* (increases with 1-*f*). In addition, the magnitudes of the speeds significantly differ between the species, where much larger average speeds are obtained for the *B. subtilis*. Figure 2D presents scattered data for the speed of both species for many *ρ* and *f*, showing that the speed of the two species is largely independent. Overall, the microscopic speeds of the mixed colony follow the same trend as the expansion speed. The average speed of the *B. subtilis* was lower compared to the axenic case, while the average speed of *P. aeruginosa* was dramatically higher. Interestingly, a mix of *B. subtilis* with an immotile mutant of *P. aeruginosa* (no flagella) have shown that the latter does not leave the inoculation area (Fig. S1), meaning that the WT *P. aeruginosa* in the mix are not being simply pushed outwards by the *B. subtilis*; they swarm slower and make it to the exterior regions of the mixed colony (similar findings of immotile cells that remain at the center of a swarm composed of a mix with WT cells was reported in^16^). The results shown here are interesting and different from those reported for immotile *B. subtilis* in^5^ because the motion of the WT *P. aeruginosa* is low—almost 0—so as far as it concerns speed, it is not so different from the immotile strain.

**Figure 3:**
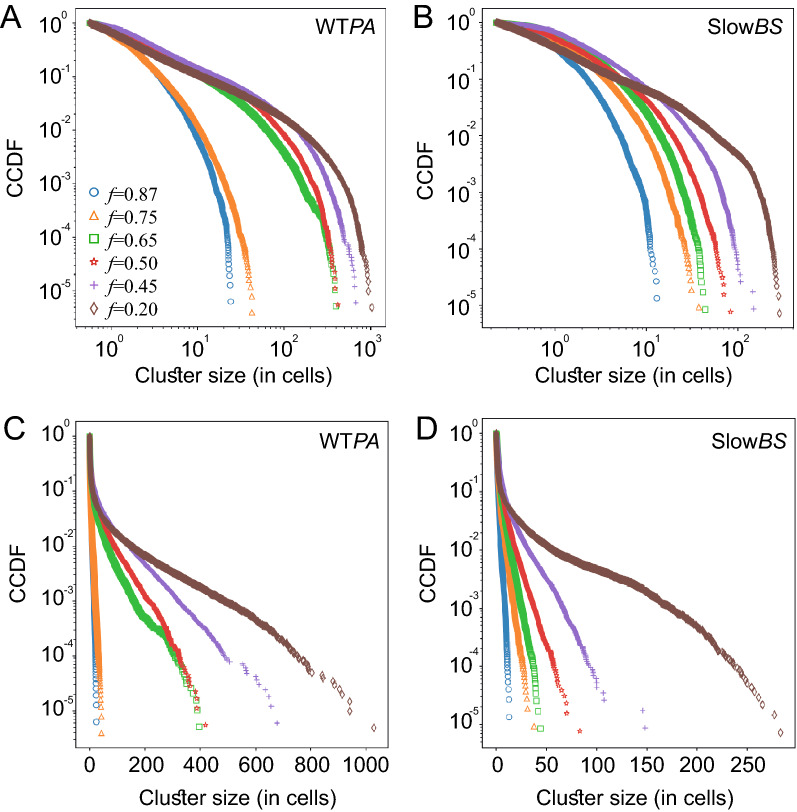
Cluster size distribution. The Complementary Cumulative Distribution Function (CCDF) of same-species cell-clusters sizes. (**A**,**C**) WT *P. aeruginosa* (mixed with WT *B. subtilis*) and (**B**,**D**) slow *B. subtilis* (mixed with WT *B. subtilis*). Top: log–log plots, compare with the bottom, showing semi-log plots. Curves show different density ratios, *f*, at a fixed area fraction *ρ* = 0.25. As expected, the characteristic cluster size decreases with *f*. With *f* = 0.2, the cluster size-distribution of slow *B. subtilis* becomes significantly wider, resembling a power-law across two order of magnitudes with an exponential cutoff.

Figure 2E, F show the spatial microscopic correlation between the two WT species in the area between the two fronts. Spatial correlations were obtained by dividing the viewing area into equally sized bins (Fig. 2E), and calculating the Pearson correlation between the area fraction of each of the two species in the bins. The results (Fig. 2F) show that the mixed species exhibit negative correlations, supporting the observation in Fig. 1C that they self-segregate into clusters and on average tend not to occupy the same bin. In addition, the data shows that the absolute value of the correlation is larger (more negative) for smaller *f*, indicating that the segregation into separate clusters is enhanced by the *P. aeruginosa*. The distribution of cluster sizes (Fig. 3A, C) show that *P. aeruginosa* clusters have an exponential distribution with a characteristic cluster size or a finite-distance effect. As expected, the characteristic cluster size decreases with *f* (at fixed *ρ* = 0.25), indicating that more *P. aeruginosa* results in larger clusters. See Supplementary text and Fig. S2 for details on the clusters’ analysis method.

## Comparison with similar swarms

In order to further study the interactions underlying the observed spatial-segregation between the species, we study several types of heterogeneous swarms and mutants with similar (or analogue) physical properties. In particular, we wish to rule out possible physical interactions as the cause of local or global segregation.

### Wild-type *B. subtilis* mixed with slow *B. subtilis*

**Figure 4:**
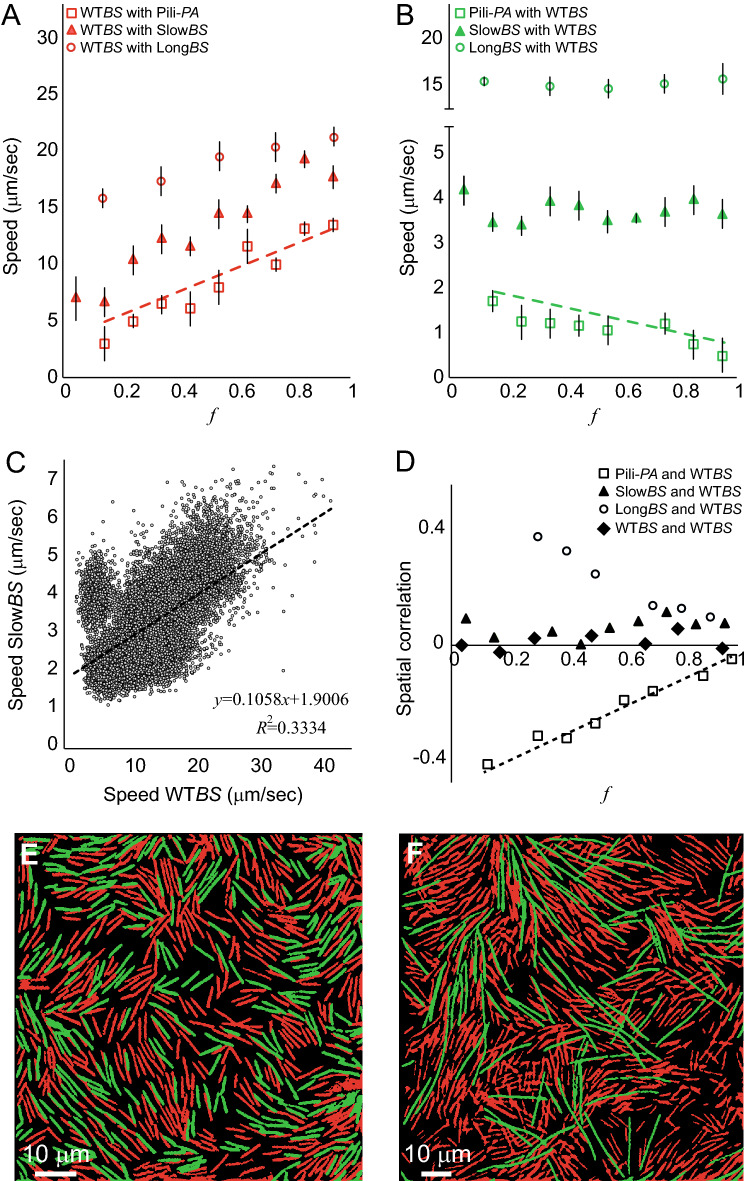
Dynamics analyses of swarm colonies composed of WT *B. subtilis* mixed with a mutant. (**A**) The average speed of the WT *B. subtilis* as a function of *f* at a fixed area fraction *ρ* = 0.25, for 3 mixed cases: pili-defective *P. aeruginosa* (open squares), slow *B. subtilis* (solid triangles), and long *B. subtilis* (open circles). All results show an increase with approximately the same slope*.* The dashed line indicates data from Fig. 2 (mixing with the WT *P. aeruginosa*). (**B**) The average speed of the mutant mixed with the WT *B. subtilis* as a function of *f* (the density ratio of *B. subtilis*). While results with WT *P. aeruginosa* show a decrease, results with the two *B. subtilis* strains do not. The dashed line indicates data from Fig. 2 (mixing with the WT *P. aeruginosa*). (**C**) The speed of slow *B. subtilis* as a function of the speed of WT *B. subtilis*, pooled overall *ρ* and *f*, shows a strong positive correlation. Note the difference compared to Fig. 2D. (**D**) The correlation in spatial distribution as a function of *f* for the 3 mixed cases: pili-defective *P. aeruginosa* (open squares), slow *B. subtilis* (solid triangles), and long *B. subtilis* (open circles). The dashed line indicates results from Fig. 2 (mixing with the WT *P. aeruginosa*). Data is shown also for the case of two WT *B. subtilis* that differ in their fluorescence color. (**E**) A microscopic view of the mixed cells with WT *B. subtilis* (green) and slow *B. subtilis* (red). Measurements show ~ 0 spatial correlation. (**F**) A microscopic view of the mixed cells with WT *B. subtilis* (red) and long *B. subtilis* (green). Measurements show positive spatial correlations.

The two WT species studied in the previous section differ in several characteristics; one of them is their microscopic speed. To verify whether the self-segregation is related to speed differences, a slower *B. subtilis* strain^17,37^, labelled red (see Table 1), was used instead of the WT *P. aeruginosa*. The WT *B. subtilis* for this experiment was labelled green. Macroscopically, the mixed colony did not show two fronts and the cells were homogenously mixed. Figure 4A, B (solid triangles) show that while the speed of the WT *B. subtilis* increases with *f* as seen for the mixed-species case, the speed of the slow *B. subtilis* strain did not follow the same trend seen for the *P. aeruginosa*. In addition, Fig. 4C shows that the speeds of the two strains are correlated. Figure 4D (solid triangles) shows that the spatial correlations are nearly 0 for all *f*, indicating that the two *B. subtilis* strains that differ in microscopic speeds do not exhibit any spatial segregation. An example for the spatial distribution can be seen in Fig. 4E. As control, results for two *B. subtilis* WT strains that differ in fluorescent color is also shown in Fig. 4D (diamonds).

In addition, the distribution of slow *B. subtilis* mixed with WT *B. subtilis* is different than *P. aeruginosa* mixed with WT *B. subtilis*. As before, Fig. 3B, D show that the characteristic cluster size of the slow *B. subtilis* decreases with *f* (at fixed *ρ* = 0.25). However, except at very small *f*, clusters consist of less than 50 cells (compared to ~ 500 with *P. aeruginosa*), indicating that these clusters are local fluctuations of areas rich in in slow *B. subtilis*. With *f* = 0.2, the cluster size-distribution of slow *B. subtilis* becomes significantly wider, resembling a power-law across two order of magnitudes (with an exponential cutoff at ~ 1000 cells). We hypothesize that this is because WT and slow *B. subtilis* clusters are mixed, creating huge connected components.

### Wild-type *B. subtilis* mixed with pili-defective *P. aeruginosa*

*P. aeruginosa* grow type-IV pili. It was shown^11^, that a mixed population consisting of WT *P. aeruginosa* and a mutant defective in type-IV pili exhibits a complex structure in which the two strains tend to self-segregate. Indeed, pili may cause stickiness between *P. aeruginosa* due to entanglement. Alternatively, they may cause elastic repulsion from *B. subtilis*. In order to verify whether the phenomenon described above is not related to pili grown on *P. aeruginosa* cells, a mutant defective in pili production was used, and mixed with WT *B. subtilis*. All the results obtained for the WT *P. aeruginosa* were obtained for the pili-defective mutant. Figure 4A, B (open squares) show the same trend, and so does Fig. 4D.

### Wild-type *B. subtilis* mixed with long *B. subtilis*

*B. subtilis* are longer than *P. aeruginosa* which may affect the dynamics and the geometry of the mixed swarm. *B. subtilis* mutants with significantly shorter lengths are not available, however, longer mutants are. In^17,34^, mixed colonies of WT *B. subtilis* and a similar elongated strain were grown. For comparison, microscopic swarming results are reproduced here. The mixed colony was macroscopically homogenous. Moreover, similarly to the slow *B. subtilis*, the speed of the elongated *B. subtilis* strain did not follow the same trend seen for the *P. aeruginosa* (Fig. 4B open circles). Most importantly, the microscopic spatial distribution showed positive correlation (Fig. 4D open circles). An example for the spatial distribution can be seen in Fig. 4F. We thus suggest that differences in lengths are not the leading reason for the clear species-dependent interactions seen here.

### Wild-type *B. subtilis* mixed with extracellular polymeric substance—defective *P. aeruginosa*

Assuming hypothetically that extracellular polymeric substance (EPS) secreted by WT *P. aeruginosa* may cause the stickiness of the cells, experiments were repeated using a double-mutated *P. aeruginosa* strain lacking two EPS genes; the pel and the psl. In their absence, swarming is thought to be enhanced as cells adhere less. However, we could not detect any qualitative difference between results obtained for the mixed colonies of *B. subtilis* and this strain, and results seen for the mix of the WTs. See Fig. S3. This suggests that EPS secretion is not a leading factor in the self-segregation observed in our mixed swarming experiments.

### Wild-type *B. subtilis* mixed with quorum-sensing—defective *P. aeruginosa*

Attraction to same-species cells or repulsion from a competing species may be achieved by positive or negative chemotaxis. Such a mechanism requires information on the local densities of the populations. To this end, three quorum-sensing deficient *P. aeruginosa* mutants were mixed with *B. subtilis* and allowed to swarm as in the above experiments. The three mutants are ΔlasR (strain 102 and strain 680 which is a GFP version), ΔrhlR (strain 103), and a double mutation ΔlasR ΔrhlR (strain 104). All mutants exhibit similar axenic swarm characteristics that are also similar to those obtained for the *P. aeruginosa* WT. In addition, the microscopic dynamics, speeds and the microscopic spatial distributions were similar to those seen for the mixed WTs. Also, results for a fourth strain with *enhanced* quorum-sensing activity (strain 847), have shown similar microscopic dynamics. The above results indicate that quorum-sensing is not a plausible mechanism in controlling self-segregation at the microscopic scale. See Fig. S3 for strains 680 and 847 that are fluorescently labelled. Results for the non-GFP strains are same, but are qualitative only. On the other hand, mixed colonies of WT *B. subtilis* with strain 102 (or 680), and WT *B. subtilis* with strain 104, expand in speeds that are similar to those obtained for WT *B. subtilis*. The latter is twice faster than the one obtained for the mixed WT *B. subtilis* with WT *P. aeruginosa* or WT *B. subtilis* with strain 103. This suggests that lasR has a role in controlling the macroscopic expansion of the mixed colony. However, lasR does not affect the macroscopic segregation (*B. subtilis* is at the front), and the microscopic dynamics shows arrested segregation.

Figure S4 shows microscopic and macroscopic images for some of the mixing cases, for instance, mixing WT *B. subtilis* with pili-defective *P. aeruginosa*; with quorum-sensing-defective *P. aeruginosa*; with quorum-sensing-enhances *P. aeruginosa*; and with EPS-defective *P. aeruginosa*. All exhibit similar patterns (the fluorescence intensity may differ due to different labelling processes).

### Biochemical interactions

**Figure 5:**
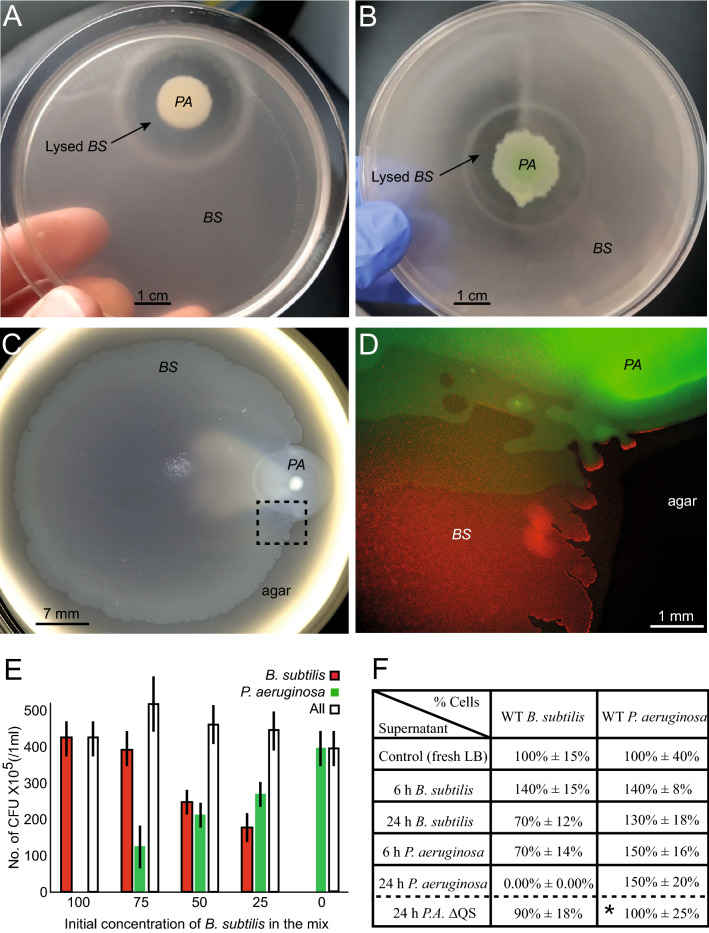
Biochemical interactions. (**A**) A 24-h old swarm plate with the two WT species initially inoculated at a large proximity (~ 5 cm). The *B. subtilis* spread throughout the entire plate, while the *P. aeruginosa* started lysing *B. subtilis* cells, forming an inhibition ring. (**B**) Same as A but for a single mixed colony. (**C**) Early stages of two WT colonies initially stated at a large proximity. No chemotaxis, attraction or repulsion was evidenced. (**D**) A fluorescence image at a larger magnification of the framed region in C, showing a mixed boundary. (**E**) Growth of the two WT species in a mixed broth for a variety of initial ratios between them. The Y-axis shows the number of cells after 6 h. Error bars represent the statistical error from 5 independent experiments. (**F**) Growth of each of the species in different media and for two different durations. The 100% for each of the species is considered as the growth in fresh LB. The last line marked with * shows results for a mix of WT *B. subtilis* and quorum-sensing deficient mutant of *P. aeruginosa* (strain 104). While *B. subtilis* grew in this mix, they did not grow when mixed with the WT *P. aeruginosa* (data above the dashed line in the table).

*B. subtilis* and *P. aeruginosa* inoculated separately (Fig. 5A), or jointly (mixed prior to inoculation) (Fig. 5B), on the same plate will eventually (> 24 h) exhibit extinction of the *B. subtilis*. However, swarm experiments are much shorter, and last about 6 h. In order to explore whether the segregation is a result of a biochemical interaction, we have first inoculated the two species in close proximity on a swarm plate and looked for attraction or repulsion between them. For all initial distances between the colonies, a 6 h exposer did not yield attraction or repulsion (e.g., Fig. 5C, D).

Next, we looked at interactions in liquid bulk. This was done by growing the two species as a mix in a liquid medium for 6 h, at a variety of initial mixing ratios (Fig. 5E). It should be noted that genes expressed by planktonic bacteria (growing in liquid medium) may differ compared to swarming ones. On the other hand, liquid medium reduces physical interactions between cells as it eliminates cell-surface interactions, cell–cell excluded volume interactions, and hydrodynamic interactions (due to constant shaking). Thus, planktonic assays allow complementary results not seen in swarm ones. We also grew each of the species for 6 h in a 6 h old supernatant of the competing species (without the presence of the cells), and for 6 h in a 24 h old supernatant of the competing species. As control, each species was grown in its own supernatant and in fresh medium for 6 h. In each experiment we counted the number of colony-forming-units (CFU) for each of the species as an indication for putative deadly bacterial competition. Our results (Fig. 5E, F) indicate that 6 h interactions in liquid do not give rise to cellular inhibition (although *P. aeruginosa* shows some increased growth in supernatants, and *B. subtilis* shows some decreased growth in supernatants). Interestingly, the only significant exception was found with *B. subtilis* grown for 6 h in a 24 h old supernatant of *P. aeruginosa* (Fig. 5E), where no *B. subtilis* cells could grow (no cells at all, in all experiments). A complementary experiment (bottom line of Fig. 5F) shows that the *B. subtilis* grows nicely in a 24 h old supernatant of a quorum-sensing defective *P. aeruginosa*, showing that the quorum-sensing is a mechanism controlling the death of *B. subtilis*. On the other hand, the segregation at the micro scale on a swarm plate with this mutant was similar to those obtained with the WT. As noted above, gene expression in supernatant may differ compared to swarming cells.

Lastly, WT *P. aeruginosa* were introduced into a *B. subtilis* supernatant (free of *B. subtilis* cells), and then allowed to grow on a swarm plate (0.22 µm filtered). The colony grew significantly faster compared to regular swarming *P. aeruginosa* but much slower than the mixed WTs colony. In addition, the cells swarmed at higher microscopic speeds, but slower than in a mixed WTs colony. Using the supernatant of a surfactin-defective *B. subtilis* strain, the *P. aeruginosa* did not exhibit the fast mode of swarming. The results suggest that the WT *P. aeruginosa* do not seem to exploit the supernatant of the *B. subtilis* in the first 6 h of contact for expansion, but do exploit the surfactant present in the supernatant.

## Discussion

Here, we showed both microscopic and macroscopic separation in dynamically swarming mixed *B. subtilis* and *P. aeruginosa* colonies. However, the two species do swarm together, forming a single colony, yet spatially heterogeneous. In particular, there is no inhibition region or demarcation line (a low-density region) between the species, as seen at much later stages (Fig. 5A, B). The differences in physical characteristics such as cell length, cell speed, the presence of pili and ability to produce EPS do not seem to affect the self-segregation between the species. Moreover, biochemical characteristics such as quorum-sensing, chemotaxis, and other potential signaling interactions in liquid media also fail to point to a plausible self-segregation mechanism. Some of our results on the biochemical interactions between the species are still poorly understood and require further study.

The swarming results can be explained in several ways. First, the control experiments, using different strains as a proxy to the other species, may not be sufficiently close to the physical or biological properties of the mixed-species swarm. For example, *P. aeruginosa* is not physically similar to slow *B. subtilis* (they are different in the number of flagella, the type and number of secreted surfactants and cell length); Moreover, the speed of slow *B. subtilis* is still higher than that of *P. aeruginosa* (Fig. 4). Similarly, mixing fast (WT) cells with slow, short cells is not physically equivalent to mixing fast normal cells with fast long cells. In addition, these effects may contribute synergistically to the spatial heterogeneity.

Alternatively, the fact that we did not succeed to obtain local segregation using different strains of the same species, may point to a different underlying inter-species interaction. Our results are consistent with a mechanism that allows cells to recognize their own species. Such species-dependent interactions are broadly defined as differential treatment (typically adverse) of cells according to their kind or type. The main reason which is typically assumed as a cause of such discrimination is competition between the species over resources. This was found, for example, in biofilms that were composed of more than one species. For example, Rosenberg et al.^8^, showed a lethal, cannibalistic example of acute competition in an interspecies interaction between biofilms of *Bacillus simplex* and *B. subtilis*. Powers et al.^7^ show inhibition of differentiation in biofilms of *B. subtilis* mixed with *P. aeruginosa*. In both cases, the “aggressive” species can modify gene expression patterns in the rival species^8^. Adverse discrimination based on species-identification was found also between sympatric expanding colonies^38^, namely, a formation of clear boundaries between neighboring swarm colonies of the same species but not the same strain; each colony grew from a different single strain (done e.g., with *B. subtilis*^39^, *P. mirabilis*^40^, *M. xanthus*^41^ and *P. aeruginosa*^42^).

In this paper, however, the swarming of *P. aeruginosa* was improved by (at least partial mixing) the two species, which is different than the prevailing competition assumption. In addition, the biochemical interactions leading to discrimination in the above-mentioned examples describe results on much longer timescales compared to those presented here. Instead, our results suggest a shift between competition and cooperation, depending on the time scale.

Several observations further support the hypothesis that some species-dependent discrimination mechanism is at play, at least in the broad meaning of the term as a differential cross-species interaction. (i) The species exhibit macroscopic colony-scale separation. (ii) Each species exhibits a larger average microscopic speed as a function of their self-partial density in the mix. (iii) Each species self-segregates into separate microscopic clusters yielding a negative correlation, and (iv) the local segregation between *P. aeruginosa* and *B. subtills* decreases with *f* (the absolute value of the correlation decreases), suggesting it is mostly due to the properties of *P. aeruginosa.* Lastly, lasR seems to affect the macroscopic expansion, but the exact biochemical origin of the phenomena we report on the microscopic scale was not identified.

## Supplementary Information

Supplementary Video 1.

Supplementary Information 1.

## Data Availability

The datasets used and/or analyzed during the current study available from the corresponding author on reasonable request.
