# Supplementary material for: Mixed-species bacterial swarms show an interplay of mixing and segregation across scales
Source: Sci Rep. 2022 Oct 3;12:16500. doi: 10.1038/s41598-022-20644-3 (PMC9529924; doi:10.1038/s41598-022-20644-3)
Supplement: Supplementary file 2 — Supplementary Information 1. [file 41598_2022_20644_MOESM2_ESM.pdf]

Quantifying cluster size distributions

We identify clusters of cells by thresholding and a connected component analysis. Thresholding is based on the intensity values obtained from the fluorescent images.

As clustered cells in the fluorescent image often appear brighter than isolated cells, the intensity of isolated cells has to be artificially increased in order to use a uniform threshold. To achieve this, we perform a preprocessing step: We check the intensity of each pixel and the average intensity of pixels in some local neighborhood. If the average intensity of pixels in the local neighborhood is below some value and the pixel intensity itself is above some threshold, the pixel's intensity is increased. This pixel-based preprocessing effectively increases the intensity of isolated cells.

Then, the image is smoothed by a Gaussian filter ($\sigma = 1$) and binarized (background=0, cells=1) by choosing an appropriate threshold. Next, connected components of pixels are identified using 4-connectivity and labeled. During this process, small clusters containing only a few pixels are disregarded as noise. Finally, cluster sizes are rescaled from pixels to cells, by dividing by the average number of pixels per cell for the respective strains or species (for this reason, the number of cells can be smaller than one). The resulting clusters are shown in Figs. S1. The surface coverage ρ_l and ρ_r of the left and right panels respectively are also determined from this computation.

Next, we consider the cluster size distribution (CSD) of the right panel as a function of the ratio between the species, *f*. Here, *f* is defined as

$$f = \rho_l/(\rho_l + \rho_r),$$

where ρ_l and ρ_r are the surface coverage of the left (always WT *B. subtilis*) and right (with WT *P. aeruginosa* or slow *B. subtilis*) panels respectively.

For each experiment, we compute the complementary cumulative distribution function (CCDF) of the clusters. Data is collected for 1000 consecutive frames (40× magnification, 50 fps) and labeled according to the averaged *f*. To take care of fluctuations in intensity, which appear naturally in such experiments, thresholds are adopted by a standard histogram matching.

See Figs. S1A-B for example analysis with WT *B. subtilis* and WT *P. aeruginosa*. See Figs. S1C-D for example analysis with WT *B. subtilis* and slow *B. subtilis*.

Supplementary figures/movies

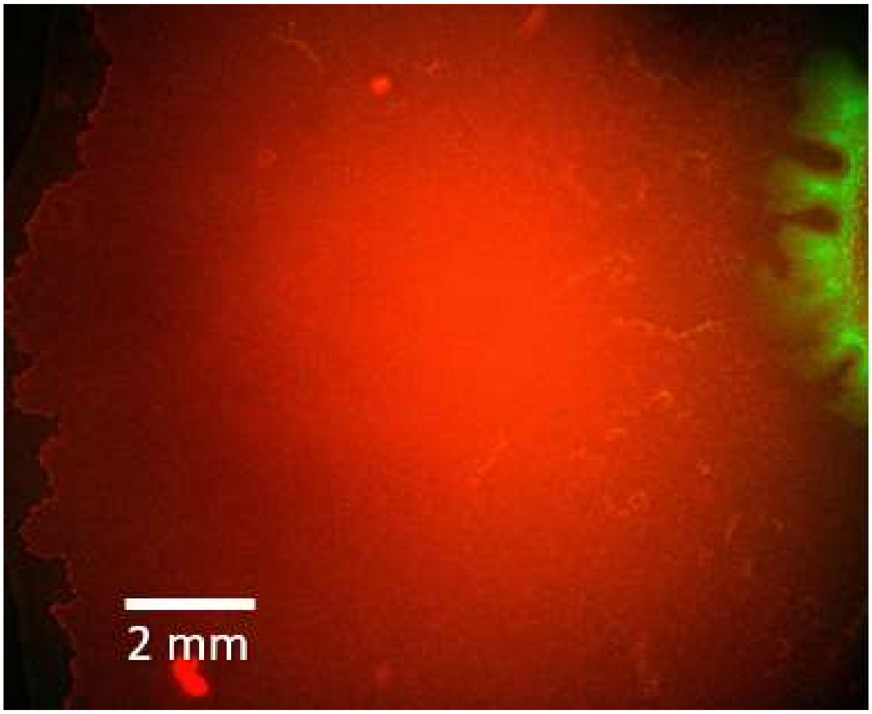

Figure S1: Mixing *B. subtilis* (red) and immotile *P. aeruginosa* (green) at a 1:1 ratio. The immotile *P. aeruginosa* cells remained at the center of the colony.

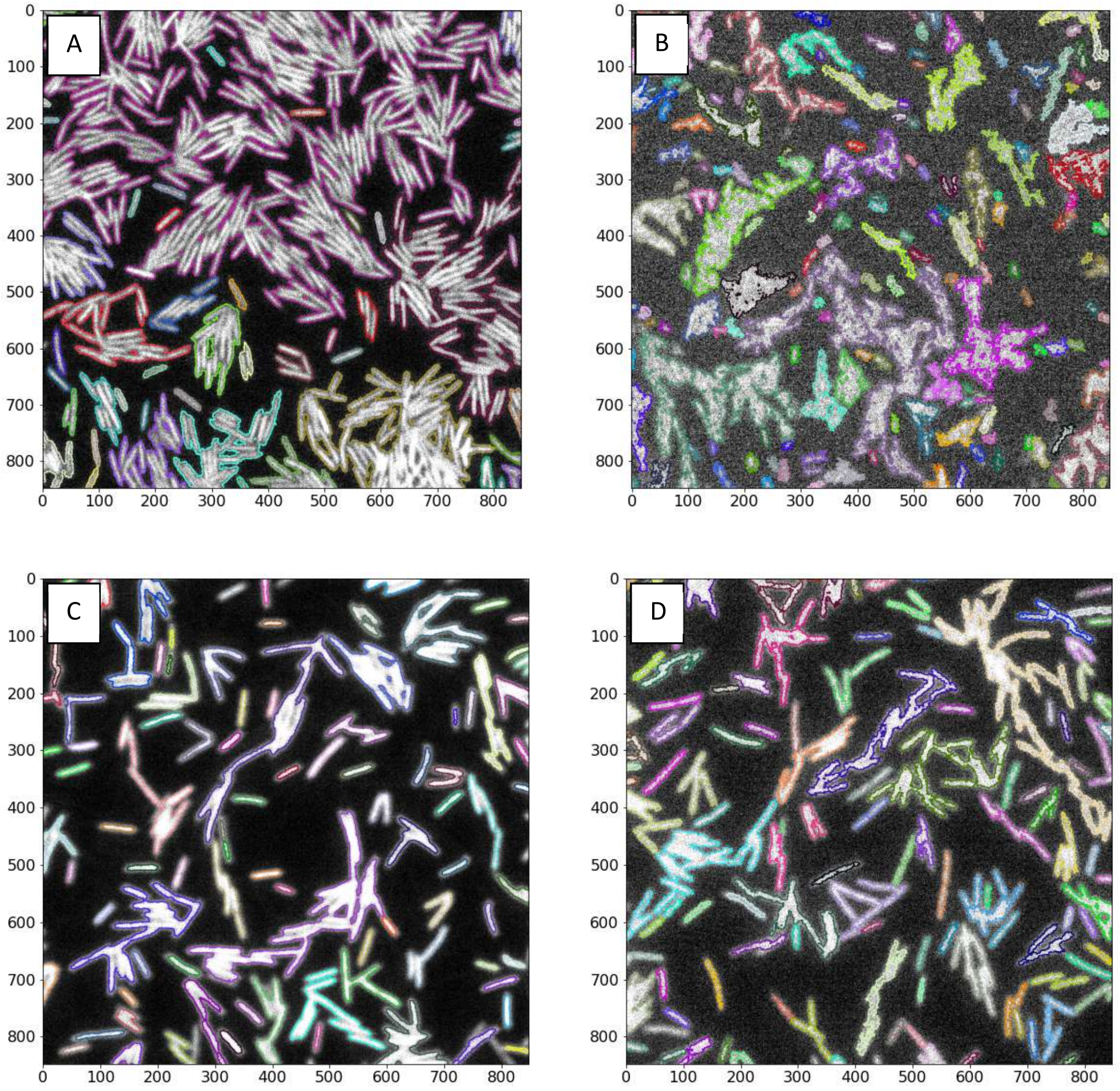

Figure S2: (A-B) Identified clusters for WT *B. subtilis* (A) and WT *P. aeruginosa* (B) for an example experiment. (C-D) Similar for WT *B. subtilis* (C) mixed with slow *B. subtilis* (D).

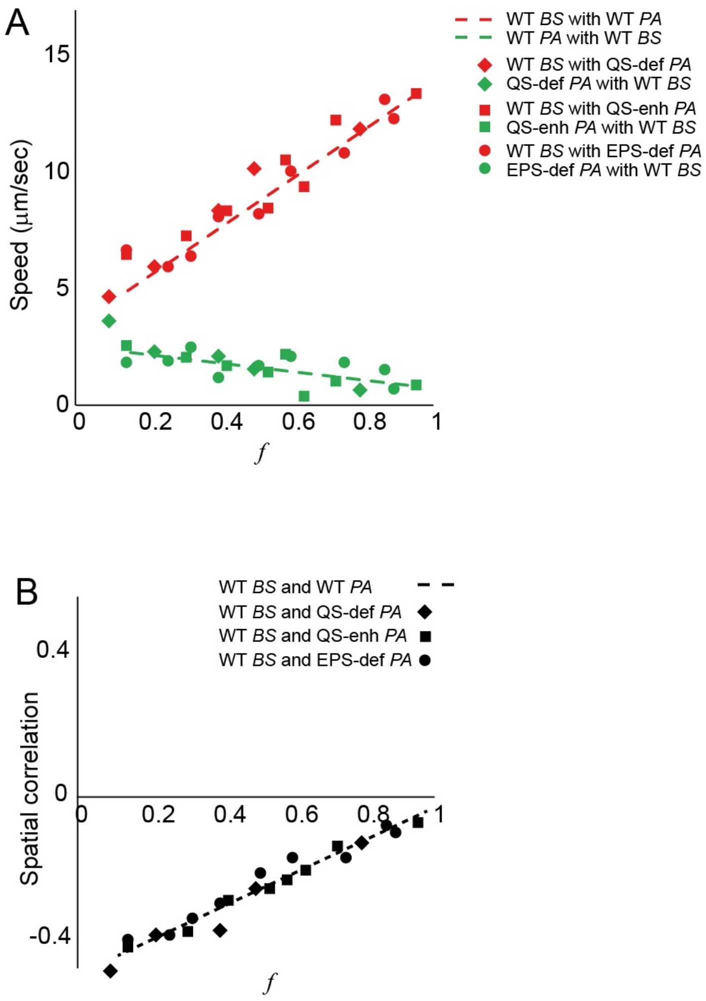

Figure S3: (A) The average speed of the WT *B. subtilis* (red) is an increasing function of the density ratio f at fixed area fraction ρ =0.25. The average speed of the different *P. aeruginosa* mutants (green) is a decreasing function of the density ratio f (the partial density of *B. subtilis*). The average is taken over different experiments and the error bars (standard deviation between experiments) are of the order of those seen in the WT case. (B) Spatial correlations as a function of f for the 3 mixed cases of WT *B. subtilis* and *P. aeruginosa* mutants. The QS strain used here is 680 (ΔlasR).

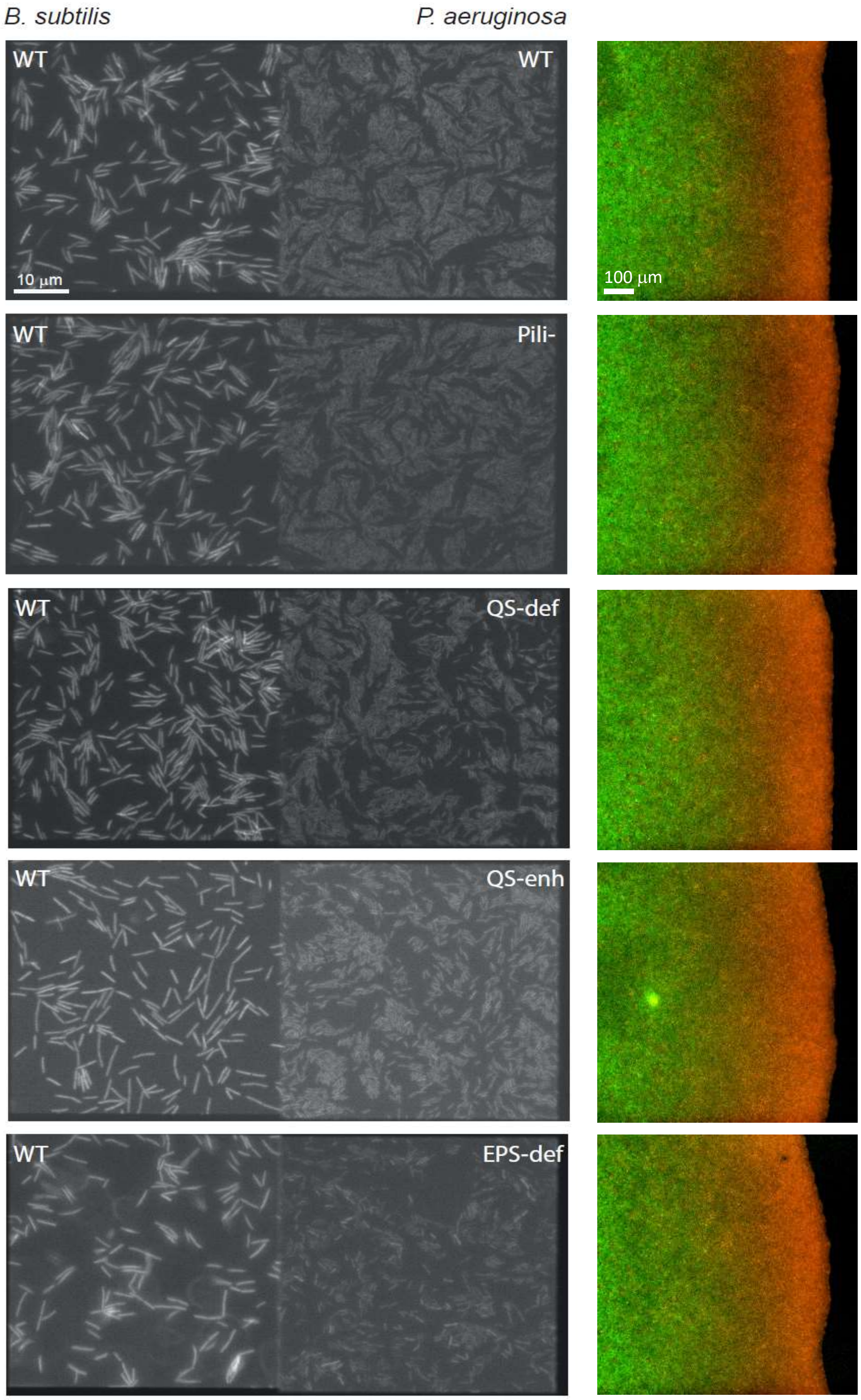

Figure S4: Microscopic view of mixed colonies. The images show the mixed region between the separated fronts. Images show the exact same spot at exact same time with different fluorescence channels (dual excitation). Left: WT *B. subtilis*. Middle: *P. aureginosa* – from top to bottom: WT, pili-defective mutant, quorum-sensing defective mutant (680; ΔlasR), quorum-sensing enhanced mutant (847; mexT) and EPS defective mutant (Δpel-Δpsl). Right panel shows the macroscopic images of the mixed colonies for each of the cases.

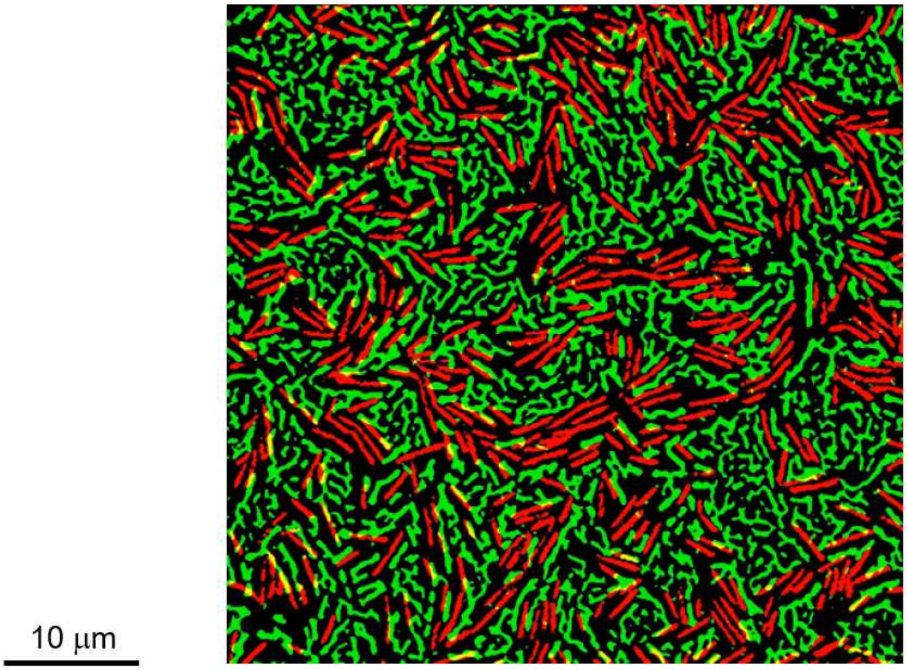

Movie S1. A real-time dual-fluorescence microscopy movie of the two WT species mixed in a swarm colony. Red cells are *B. subtilis* and green cells are *P. aeruginosa*. The frame size is 100 μm.
